# A holistic model of health inequalities for health policy and state administration: a case study in the regions of the Czech Republic

**DOI:** 10.1186/s12939-023-01996-2

**Published:** 2023-09-05

**Authors:** Dana Hübelová, Jan Caha, Lenka Janošíková, Alice Kozumplíková

**Affiliations:** 1 Department of Social Studies, Mendel University in Brno, Zemedelska 1, Brno, 613 00 Czech Republic; 2 Department of Regional Development, Mendel University in Brno, Zemedelska 1, Brno, 613 00 Czech Republic

**Keywords:** Determinants of health, Regional disparities in health, Spatial differentiation of health, Health promotion and prevention, Healthcare system optimization

## Abstract

**Background:**

Health inequities exist within and between societies at different hierarchical levels. Despite overall improvements in health status in European Union countries, disparities persist among socially, economically, and societally disadvantaged individuals. This study aims to develop a holistic model of health determinants, examining the complex relationship between various determinants of health inequalities and their association with health condition.

**Methods:**

Health inequalities and conditions were assessed at the territorial level of Local Administrative Units (LAU1) in the Czech Republic. A dataset of 57 indicators was created, categorized into seven determinants of health and one health condition category. The necessary data were obtained from publicly available databases. Comparisons were made between 2001–2003 and 2016–2019. Various methods were employed, including composite indicator creation, correlation analysis, the Wilcoxon test, aggregate index calculation, cluster analysis, and data visualization using the LISA method.

**Results:**

The correlation matrix revealed strong relationships between health inequality categories in both periods. The most significant associations were observed between Economic status and social protection and Education in the first period. However, dependencies weakened in the later period, approaching values of approximately 0.50. The Wilcoxon test confirmed variations in determinant values over time, except for three specific determinants. Data visualization identified persistently adverse or worsening health inequalities in specific LAU1, focusing on categories such as Economic status and social protection, Education, Demographic situation, Environmental status, Individual living status, and Road safety and crime. The health condition indices showed no significant change over time, while the aggregate index of health inequalities improved with widened differences.

**Conclusion:**

Spatial inequalities in health persist in the Czech Republic, influenced by economic, social, demographic, and environmental factors, as well as local healthcare accessibility. Both inner and outer peripheries exhibit poor health outcomes, challenging the assumption that urban areas fare better. The combination of poverty and vulnerabilities exacerbates these inequalities. Despite the low rates of social exclusion and poverty, regional health inequalities persist in the long term. Effectively addressing health inequalities requires interdisciplinary collaboration and evidence-based policy interventions. Efforts should focus on creating supportive social and physical environments, strengthening the healthcare system, and fostering cooperation with non-medical disciplines.

**Supplementary Information:**

The online version contains supplementary material available at 10.1186/s12939-023-01996-2.

## Background

Health inequalities are viewed as unfair differences resulting from a large number of determinants often of a very different nature [1]. Ideally, every individual should have an equal opportunity to reach their full health potential, and no one should be disadvantaged in achieving it if such disadvantage can be avoided [2]. Health inequalities that could be prevented by appropriate means are perceived as the result of inequities in society [3]. These inequalities begin at birth [4] and are largely shaped by socioeconomic determinants over the course of one’s life. These are the conditions in which people are born, grow up, live, work and age [5]. In general, real conditions are shaped by the distribution of finance, energy and global resources at the national and local levels. Health inequalities are caused by government policies affecting the quantity, quality and distribution of determinants and are also influenced by policy decisions [6].

A body of research demonstrates that preventable systematic inequalities in health exist both between and within societies, and at all hierarchical levels [7–11]. Health inequalities are also spatial between regions, urban and rural areas, and within urban areas [12, 13]. While the overall level of health in European Union countries has been improving in recent decades, significant disparities remain for people living in socially, economically or societally disadvantaged conditions [14].

Given the multitude of factors that influence the emergence of health inequalities, it is desirable that the assessment of the determinants is as comprehensive as possible. This leads us to the idea of forming a holistic concept of health inequalities, even though the holistic approach is more commonly associated with health as such. The holistic approach is closely associated with the concept as emphasized in the study [1], particularly concerning the approach to the patient and the need to develop tools for systematic healthcare application. By applying this approach, our goal is to contribute to the evaluation of health inequalities through a complex and detailed assessment at the local level. We draw on earlier comprehensive health studies, where the Canadian Health Report “*A new perspective on the health of Canadians”* [15] can be considered the first comprehensive conception of health. The study [1] proposed a conceptual framework for the social determinants of health, including four highly interrelated categories. The categorization of determinants of health has also been used in other studies [16, 17]. The study, which presents a comprehensive perspective on the factors influencing human health [18], merged the categories from other studies [17, 19] to create a broad framework for defining a holistic conceptualization of the determinants of health. However, these determinants are not the same in relation to individual health potential. The study proposed a three-level system with behavioral, social and environmental categories. Determinants in each category (layer) may interact with each other and may interact independently but also collectively with determinants of other categories. Some determinants may be influenced by personal decisions, others by the economic situation or political reform [18].

### Context of health inequalities and research objectives

Several classifications of the determinants of health inequalities and their impact on population health are well-known (refer to, for example, the Conceptual Framework for Action on the Social Determinants of Health [20]). The influence of various factors on population health has been identified as follows: the genetic basis accounts for 10–15%, health and healthcare contribute 10–15%, the environment contributes 20%, and lifestyle factors contribute 50% [21]. Additionally, the County Health Ranking Model [22] uses the following proportions: health and healthcare contribute 20%, the environment contributes 10%, social and economic factors contribute 40%, and lifestyle factors contribute 30%. The Euro-Healthy project [14] produces a population health index (PHI) for EU countries at the NUTS2 level (regional level unit for the application of regional policies) and to 10 selected metropolitan areas. The results show that systematic spatial inequalities persist in Europe at the NUTS2 level. In a spatial context, the study carried out in France [13], which presents the Geographical Classification for Health Studies (GeoClasH) is inspiring and thought-provoking due to its focus on the municipal scale while assessing variables from the physical environment, social characteristics of population, and spatial accessibility to healthcare.

Our baseline study on health inequalities [2] was based on a comprehensive systems analysis in which we formed a methodological and analytical framework to integrate social, economic, demographic, health, environmental, and individual determinants of health inequalities. We created an extensive dataset and visualizations that are available online [23]. This framework respects a holistic health determinants model for public health [1, 18]. The intention of our outputs was to support policy decisions and target-selective health intervention and prevention in the Czech Republic [2]. The framework is modular and scalable. We also applied different methodological approaches to spatiotemporal analysis and comparison of results [24].

The intent of the present paper is to extend the original methodological and analytical framework of health inequalities to include the spatiotemporal dimension and the context of geographical classification. The aim of the presented study is to use the example of regions LAU1 (Local Administrative Units, level 1) of the Czech Republic to: 1) evaluate the determinants of health inequalities in space and time, 2) determine the relationship between the categories of determinants of health inequalities and their association with health condition, and 3) develop a holistic health determinants model for public health and test its objectivity in assessing health conditions.

### The starting point for the concept of health inequalities

In this paper, we build upon the results of the health inequalities assessment [2, 24]. To comprehensively record, analyze, and interpret health inequalities, it is necessary to have the broadest possible set of determinants for these inequalities. Therefore, in determining health inequalities, we start from the original concept of a holistic understanding of health, considering not only genetic and environmental factors but also extending it with additional categories. We divided contextual risk determinants into seven categories (see Appendix 1 with the List of determinants of health and health condition for more details):

- A.1 Economic status and social protection (theme Employment rate and Economic conditions and social benefits)
- A.2 Education (theme Educational structure)
- A.3 Demographic situation (theme Migration, Aging and Urbanization)
- A.4 Environmental status (theme Air quality and Countryside)
- A.5 Individual living status (theme Living condition and Technical infrastructure)
- A.6 Road safety and crime (theme Traffic accidents and Crime)
- A.7 Sources of health and social care (theme Health and social care capacities)

Category A.1 Economic status and social protection: Socioeconomic conditions are considered to be an objective cause of spatial variation in health outcomes (e.g., [25–29]), although their importance is debated and the evidence is not entirely consistent [30, 31]. Nevertheless, socioeconomic characteristics are a useful differentiator of differences in health status (or mortality; [32]). Economic status can be indirectly measured by (un)employment rates. Long-term unemployment ranks as a highly stressful life event that affects not only psychological but also physical health [33]. The social category has been studied, for example, by using indices of deprivation, which are mainly used in social epidemiology [34–36].

Category A.2 Education: Education has a significant impact on spatial and hierarchical differentials in health inequalities, as well as morbidity and mortality. One disadvantage of formal education indicators is that they fail to capture the socioeconomic positions of adults [20]. Nevertheless, education remains an input factor for the future structure of occupation and income [37]. Individuals with lower educational attainment are known to die earlier than those who are more educated [26, 38]. Moreover, knowledge and skills acquired through education also influence cognitive ability, health literacy, and health-promoting lifestyle choices [39].

Category A.3 Demographic situation: The age structure of the population shapes the current health status of the population and will also influence the future situation, including the types of population health interventions [40]. In the context of demographic aging, there will be an increase in polymorbidity and the prevalence of chronic diseases, especially cardiovascular diseases and degenerative diseases of the nervous system. These conditions are associated with overall health status and the need for outpatient and inpatient care [41, 42].

Category A.4 Environmental status: This category includes studies on the external environment that focus on exposure to various environmental components, such as air pollution [43], noise [44], water contamination [45], ultraviolet radiation [46] or green spaces [47–51]. For the assessment of Environmental status, we chose a combination of air quality indicators and the coefficient of ecological stability. The coefficient represents the proportion of ecologically stable areas, typically including green and blue areas, to unstable areas.

Category A.5 Individual living status: This category includes factors such as housing quality and technical infrastructure, which are considered important contributors to (social) inequalities in health [3, 19]. The relationship between the size of living space and subjective well-being is generally considered to be positive. The quality of housing indicator has been used as one of the quality-of-life indicators, for example in [52, 53].

Category A.6 Road safety and crime: This category serves as an indicator of inequality, as road accidents are not solely caused by driver error but are influenced by multiple factors, including road users, vehicles, transport infrastructure, and the surrounding environment [54, 55]. Crime, on the other hand, is a socially determined phenomenon influenced by various factors, such as the level of social control, sense of community [56] or income inequality [57].

Category A.7 Sources of health and social care: The availability and accessibility of health care services are generally improving, although studies indicate lower health care utilization in regions with lower density and availability of medical care [58–60]. There is an increasing interest in utilizing social care data, including sources of social care, as the demand for both quantity and quality of care rises due to population aging. This necessitates enhancing decision-making processes and transforming public services [61]. Furthermore, it is crucial to gain a better understanding of social service delivery at the local level [62].

We examined the impact of categories A.1 to A.7 on the health condition identified as category B.1. The health condition indicators we included were life expectancy by age and sex, which serves as a comprehensive indicator of mortality intensity. In a broader context, life expectancy is considered an indicator of quality of life as it reflects social and economic conditions [63–65], educational attainment [66], and the quality and availability of public health and healthcare infrastructure [67]. Health status indicators, represented by the mortality structure based on the most common causes of death, reproductive health indicators (abortion rate and maturity of a child at birth) and incidence of diabetes are influenced by socioeconomic and demographic determinants [68].

## Material and methods

**Fig. 1:**
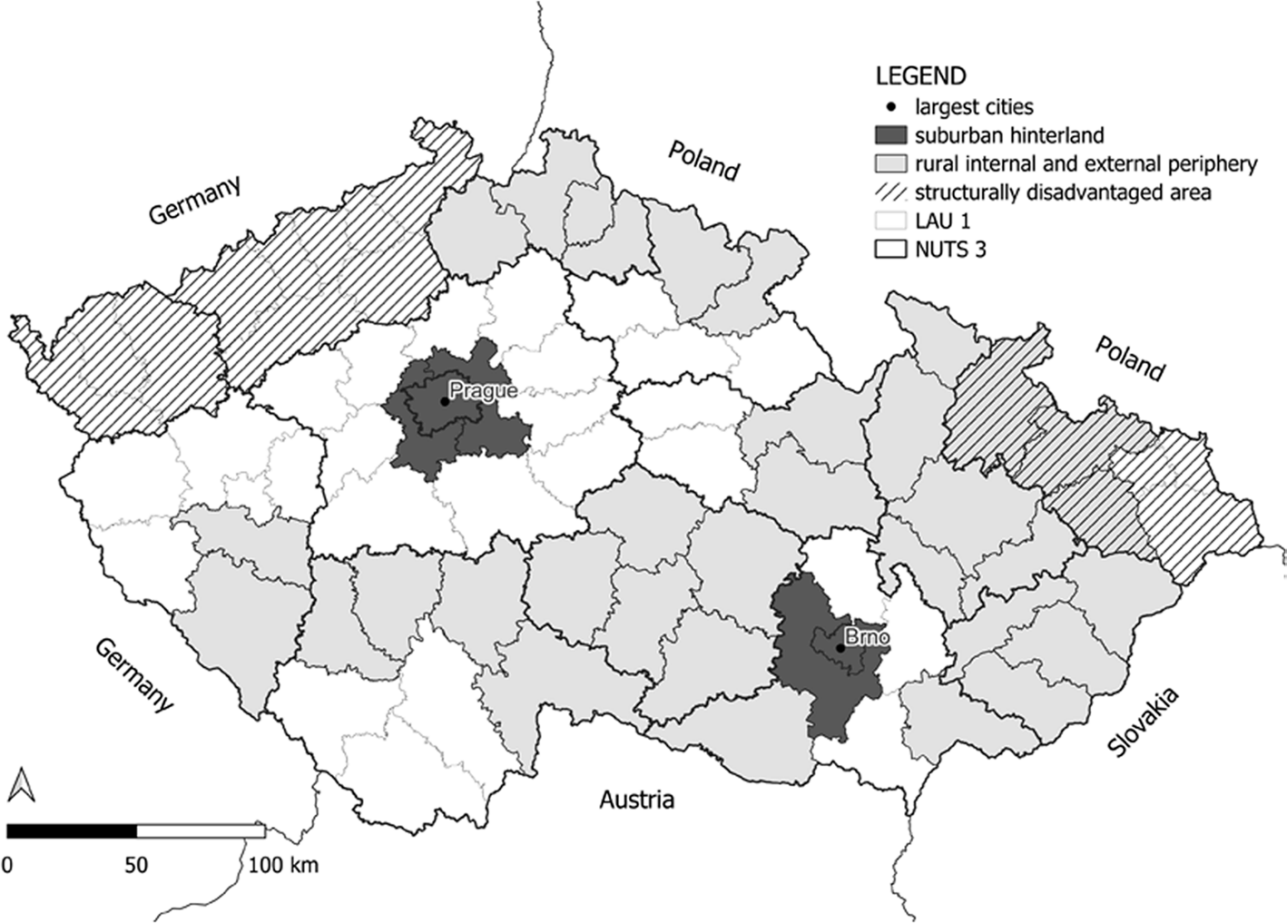
Czech Republic urban and periphery map based on the RDS CZ 2021 +

Spatial differentiation of determinants of health inequalities and health condition was assessed at the territorial level of LAU 1 (Local Administrative Units) in the Czech Republic, which consists of 76 units and the capital city of Prague. The Czech Republic is characterized by a significant fragmentation of the settlement structure and an inconsistent urban network. Differences in the settlement structure, as well as the level of urbanization, are evident in the distribution of the population into size categories of municipalities and the average size of municipalities, which varies significantly across regions. While four LAU1 (NUTS 4) units are directly formed by large cities, highly urbanized LAU1 units are predominantly found in regions with structural challenges. Conversely, the suburban hinterland of large cities lacks representation of any major cities. Figure 1 shows the spatial distribution of urban and rural areas in the Czech Republic, including Prague, the capital city, and Brno, the second-largest city, along with their suburban hinterland. The grey areas represent rural peripheries, typically characterized by inferior locational factors (such as transport accessibility and access to services) and socio-economic indicators (e.g., higher unemployment rates, limited job opportunities, negative population growth, an aging population, etc.). These rural peripheries encompass both internal peripheries within the country and external peripheries located along the borders with neighboring countries (Germany, Poland, Slovakia, and Austria). The hatched areas represent regions with historical structural disadvantages, previously focused on industrial activity.

A dataset was created for each region, comprising 57 indicators. These indicators were divided into seven categories (A.1 to A.7) with a total of 33 health determinants, and one category for health condition (B.1) with 24 health indicators (see Appendix 1). The choice of the LAU 1 territorial level was practical, as it allowed us to gather all the necessary data, even though it lacks legislative support in the Czech Republic. Higher territorial units (NUTS2 and NUTS3) have a legal basis but are geographically and economically fragmented in the Czech Republic, making them less significant for our detailed assessment. We obtained data from various publicly available databases (CZSO: Czech Statistical Office, IHIS: Institute of Health Information and Statistics of the Czech Republic, MoLSA: Ministry of Labour and Social Affairs, and CHMI: Czech Hydrometeorological Institute). Our analysis covers two distinct periods: 2001–2003 and 2016–2019, chosen based on data availability. The first period (2001–2003) was selected because it provided data for all the chosen indicators in connection with the implementation of the Census in the Czech Republic. The second period (2016–2019) was chosen to utilize the most up-to-date data available at the time of the research.

The data analyzed corresponds to these specified periods, which were selected due to the unavailability of data for a single matching calendar year (the specific year for which the data was available is stated for each indicator in Appendix 1). Two exceptions exist: 1) for variables related to Education, data from the 2011 Census had to be used within the second period; and 2) for variables related to air pollution, data from five-year averages (2007–2011) had to be utilized within the first period.

To facilitate interpretation of the results, a composite indicator (index) was created for each category (A.1 to A.7 and B.1). This index combines multiple variables mathematically and ranges from 0 to 1, with higher values indicating better outcomes. We employed the WSA (Weighted Sum Approach) method, a weighted sum method based on utility maximization principles, to calculate these composite indicators. This method assumes linearity and maximization of all partial utility functions, obtained by normalizing the original input data. The WSA method is based on 3 phases. In the first phase, the evaluation of LAU1 was obtained according to each categories A.1 to A.7 and B.1 (as health condition index) separately and with the equal weights of criteria. In the second phase the same method was used for the complete categories A.1 to A.7 together and with equal weights. This result could be taken as the aggregate index of determinants of health inequalities of each district (see [24] for more details on the methods). In the WSA method criteria can be minimized or maximized. Two formulas could be applied for the data normalization – formula (1) for maximization type and (2) for minimization criteria type:

1 $${r}_{ij}=\frac{{y}_{ij} -{ A}_{j}^{-}}{{A}_{j}^{+}- {A}_{j}^{-}}$$

2 $${r}_{ij}=\frac{{A}_{j}^{+} - {y}_{ij}}{{A}_{j}^{+} - {A}_{j}^{-}}$$

The final ranking is based on the utility – the higher is the better:$u\left({a}_{i}\right)=\sum_{j=1}^{k}{v}_{j}{r}_{ij}, \forall i=1, \cdots , p.$

We used Pearson's correlation coefficient for correlation analysis to identify relationships between the categories of health inequalities. The correlation coefficient varies between + 1 through 0 to -1, the closer the value of the correlation coefficient is to one or minus one, the stronger the relationship. Values around zero indicate that the variables have no relationship. Positive values indicate that as one variable increases, the other variable also increases. Negative values indicate that as one variable increases, the other variable decreases. This analysis explored the correlation among categories A.1 to A.7, as well as the correlation between these categories and the health condition category B.1, examining their changes over time. The Wilcoxon test, which assesses the goodness of fit of the mean for data that may not have a normal distribution, was used to test the change in values of individual determinants in all categories. A result below 0.05 indicates a significant change in values over time.

Subsequently, the sub-indexes of categories A.1 to A.7 were utilized to calculate an aggregate index, which provides a single numerical value assessing all determinants of health inequalities. This aggregate index is also employed in the cluster method. Cluster analysis is a multivariate statistical method, working with a large number of variables. An agglomerative clustering was used, the main task of which was to divide the file into several sub-files containing elements with similar variable values. The aim is to maximize inter-cluster variability while minimizing intra-cluster variability. Clustering was carried out as hierarchical, when clusters are created gradually, in individual steps. Distance measurements using a square of Euclidean distance were used to assess the similarities between clusters: $\sqrt{{(x}_{1}- {x}_{2}{)}^{2}+{(y}_{1}- {y}_{2}{)}^{2}}$.

Clusters were created using the Ward method, which uses variance. For each formed cluster, we calculated the z-scores of the determinant categories A.1 to A.7 by linearly transforming the original measured values. The z-score helps express the position of individual indices relative to the entire set. A positive sign indicates an observed value above the mean, while a negative sign signifies a value below the mean.

To visualize the data, we employed cartograms generated using the LISA (Local Indicator of Spatial Association) method, which identifies clusters with similar or different values, as well as spatial outliers. The cartograms (Figs. 3, 4, and 7) use a bivariate legend displaying low values for both indicators in the lower left corner and high values for both indicators in the upper right corner. The hatching in the legend illustrates intervals that are not present in the cartogram. For presenting results that are not primarily spatial in nature, we utilized tables and graphs.

## Results

**Fig. 2:**
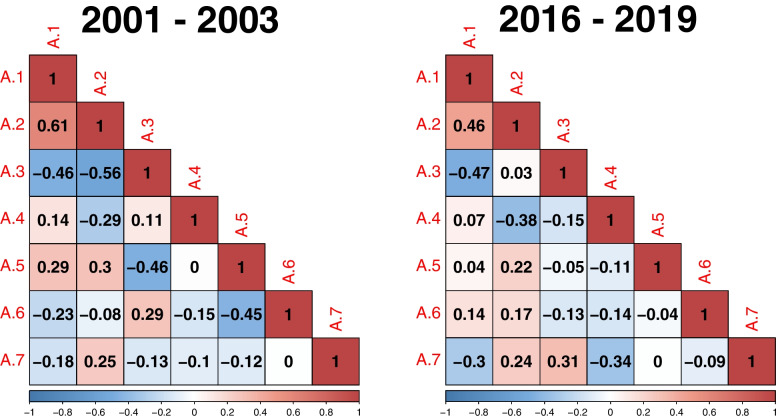
Correlation matrix of each category of health inequalities in period 2001–2003 and 2016–2019

The correlation matrix illustrates the final Pearson correlation coefficient among each category of health inequalities during the periods of 2001–2003 and 2016–2019 (see Fig. 2). In the initial period 2001–2003, the strongest relationship is observed between the categories A.1 Economic status and social protection and A.2 Education, with a correlation coefficient of 0.61. Additionally, a significant correlation is found between A.2 Education and A.3 Demographic situation, with a coefficient of -0.56. In the later period of 2016–2019, all dependencies weaken, and the most intense ones approach a coefficient of 0.50, with both positive and negative dependency values. Specifically, the correlation between A.1 Economic status and social protection and A.2 Education is 0.46, while the correlation between A.1 Economic status and social protection and A.3 Demographic situation is -0.47 (see Fig. 2).

The Wilcoxon test tested whether there is a change in the values of each determinant over time (2001–2003 and 2016–2019). The results of the test indicated that all values were found to be less than 0.05, suggesting that they do indeed vary over time. However, there were three exceptions where the values were greater than 0.05. These exceptions included the proportion of job seekers with primary education, the population per 1 physician, and standardized mortality due to liver disease.

**Fig. 3:**
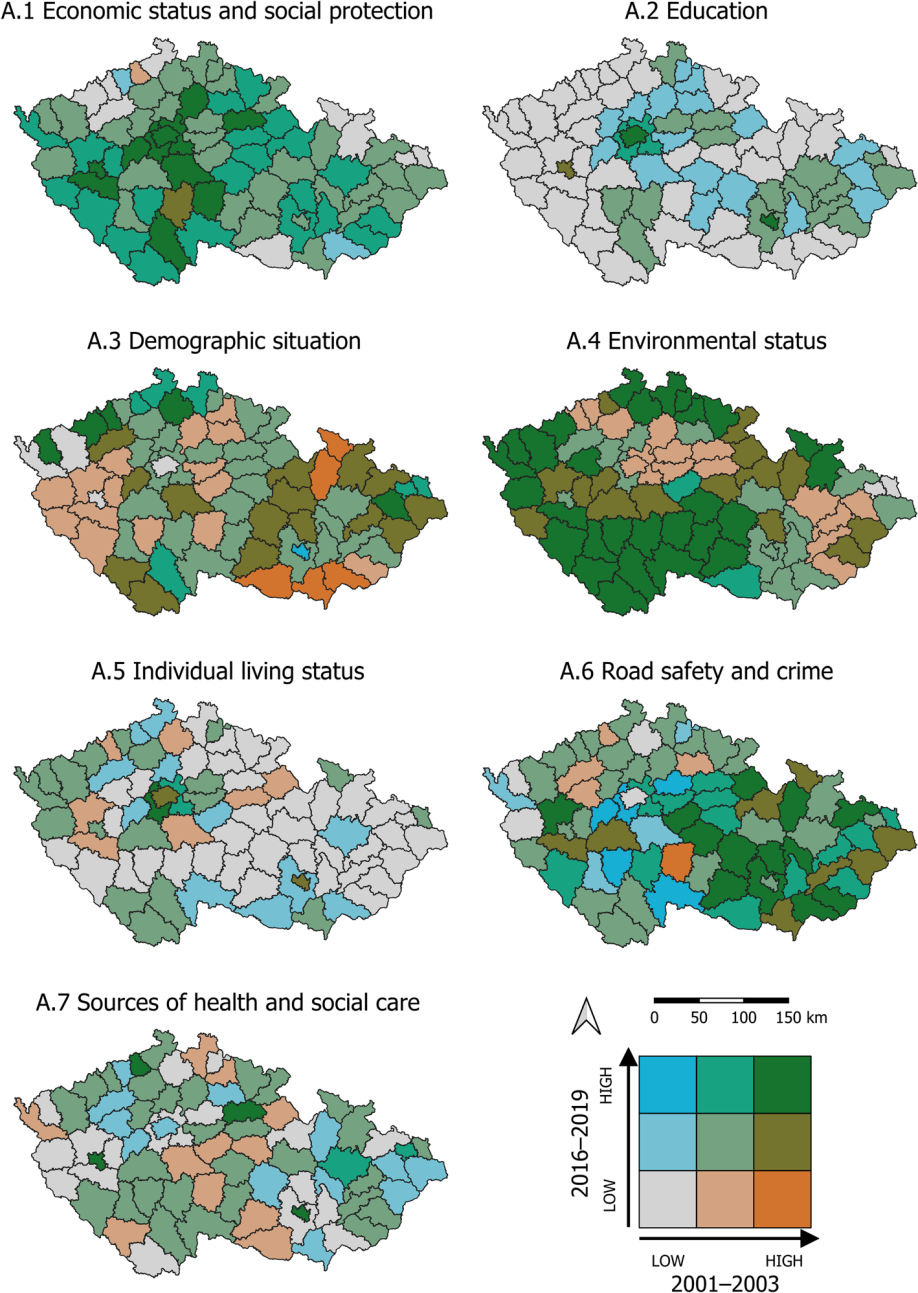
Spatiotemporal change of health determinants of categories A.1 to A.7

In our research, the spatiotemporal framework plays a crucial role, particularly in identifying the LAU1s that exhibit the most significant inequalities in health within the studied categories (Fig. 3). In the following commentary, we specifically focus on the LAU1s where the assessed categories of health inequalities consistently show adverse or worsening trends over time. For these particular LAU1s, we provide detailed information about their geographical context (Table 1).

- Category A.1 Economic status and social protection, as well as A.2 Education, consistently remain below average in LAU1s located in both the outer (border) and inner periphery (within NUTS3 administrative boundaries). The values in these regions either remain stable or worsen over time.
- In category A.3 The demographic situation, we observe values that are below average or worsening in the regions of the outer and inner periphery. Additionally, the capital city of Prague (LAU1) exhibits similar patterns.
- Category A.4 Environmental status shows a deterioration in fifteen LAU1s located in lowland areas. Furthermore, regions focused on extractive and downstream industries consistently exhibit below-average environmental status.
- A.5 Individual living status remains below average in LAU1s characterized by a predominantly rural settlement pattern and those located in the northeastern border. Moreover, these values do not significantly change over time.
- A.6 Road safety and crime indicate below average values in a small proportion of urbanized LAU1s. Additionally, only five regions have experienced a deterioration in road safety and crime over time.
- Category A.7 Sources of health and social care typically demonstrate below-average values in LAU1s that are closely adjacent to core regions. This is due to the hierarchical organization of the health and social care system in the Czech Republic. In some LAU1s located in the inner periphery (at NUTS3 administrative boundaries) or the border periphery, this system has deteriorated.

**Table 1:** Characteristics of regions with unfavorable/deteriorating outcomes according to categories of the determinants of health inequalities

Categories of health inequalities	LAU1 CZ location and basic characteristics (comparison 2001–2003 and 2016–2019)
A.1 Economic status and social protection and	Urbanized outer (border) periphery: structurally affected LAU1 with formerly intensive coal mining and associated industries; economic development in the era of industrialization
A.2 Education	Rural outer and inner periphery: mountainous border settlements after World War II; inner (administrative) periphery at NUTS3 borders: "remote peripheral" rural areas with a weakened indigenous economic base without variant economic activity, selective migration accompanied by a negative educational structure, aging population and above-average unemployment
A.3 Demographic situation	Rural outer and inner periphery with selective migration exacerbating population aging Some large cities (especially the capital Prague) with natural population decline and an above-average share of the post-reproductive population
A.4 Environmental status	Lowlands with intensive agriculture and road backbones Structurally affected LAU1 in the north-western borderlands with mining, chemical and energy industries
A.5 Individual living status	Rural outer and inner periphery, especially hills and uplands: fragmented rural settlement pattern, large number of small and very small settlements (up to 200 inhabitants)
A.6 Road safety and crime	Urbanized LAU1
A.7 Sources of health and social care	Suburban rural: in the hinterland of large regional towns with intensive suburbanization processes: the development of residential function, health and social infrastructure is provided by core catchment areas (large towns)

The composite indicator B.1 Health condition index was calculated for each LAU1 region and for each period. The values of the B.1 Health condition index did not exhibit significant changes between the periods of 2001–2003 and 2016–2019 across the entire set of LAU1 regions (see Table 2). Furthermore, the spatial differentials in the health condition index remained unchanged. In both compared periods, LAU1s with below-average health condition were identified, including structurally affected regions such as West Bohemia and the regions of the northeastern border periphery. Conversely, LAU1s with above-average health condition were found in the capital city of Prague and its suburban hinterland, the north–south central belt, and LAU1s in the southeastern border region.

**Table 2:** Descriptive statistics: health condition index and determinants of health inequalities (LAU1 CZ)

		**2001–2003**	**2016–2019**
B.1 Health condition index	Minimum	0.30	0.32
	Maximum	0.77	0.78
	Mean	0.56	0.59
	Median	0.56	0.60
A.1 – A.7 Index categories of health inequalities	Minimum	0.33	0.34
	Maximum	0.54	0.57
	Mean	0.42	0.44
	Median	0.41	0.43

**Fig. 4:**
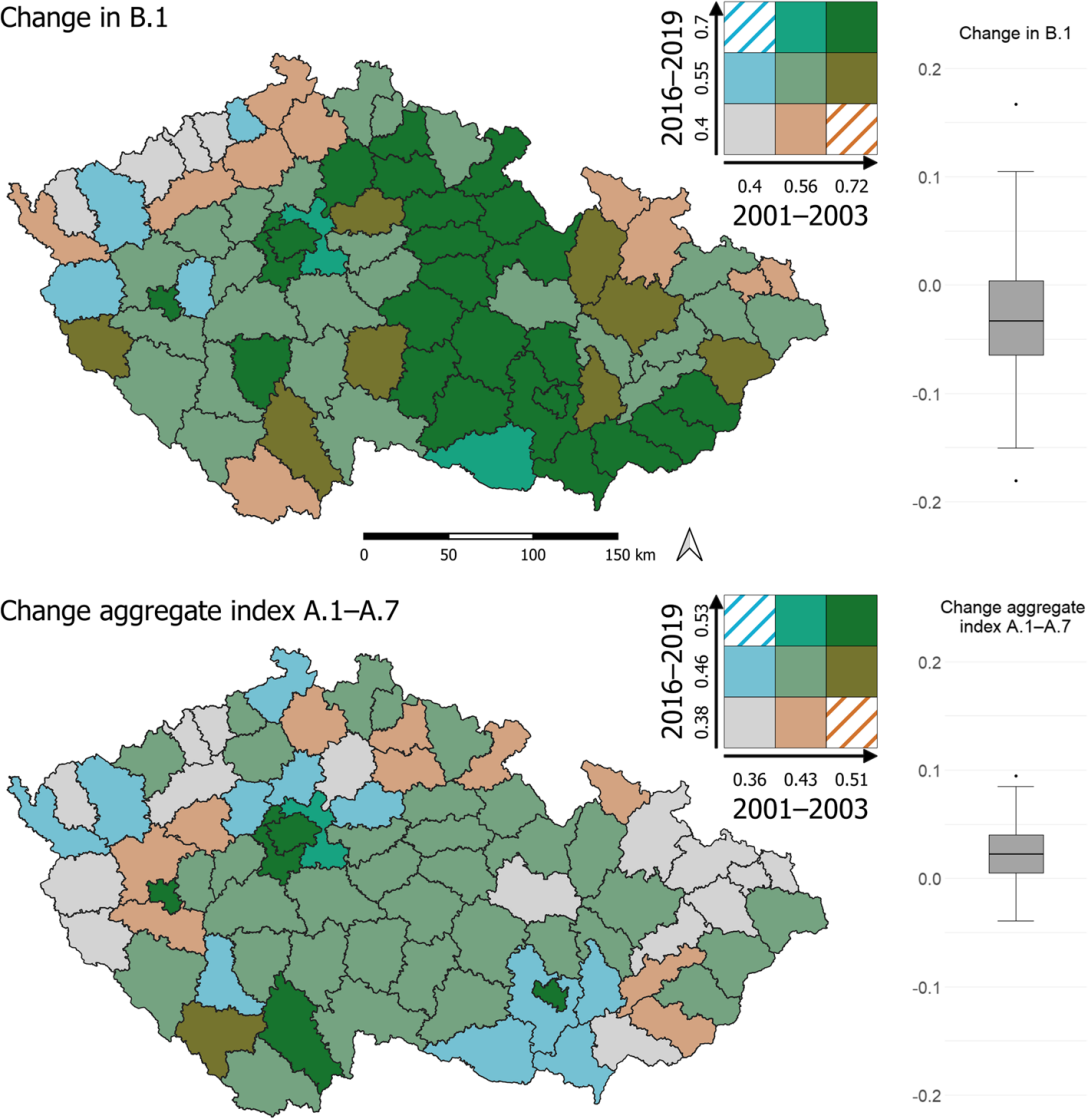
Change in health condition and aggregate index categories of determinants of health inequalities

In the case of the Health condition index, the change over time and space is not particularly positive. Although the value of this index for many LAU1 regions was already relatively high in the first observed period, it did not improve compared to the second period. Out of the total number of LAU1s, 56 (73%) experienced a decline in health condition over time, while only 21 (27%) showed improvement. It should be emphasized that the negative changes observed over time are very slight, with a difference of 0.032 for the mean and 0.033 for the median between 2016–2019 and 2001–2003, but they are supported by the fact that most LAU1s are situated in the negative portion of the "box" section of the chart, indicating a predominance of negative trends in health condition (Fig. 4; Change in B.1).

Moving on to the aggregate index of the categories of determinants of health inequalities, the values improved very slightly between 2001–2003 and 2016–2019 (Table 2), but the differences between the values widened in LAU1 regions. In the case of the aggregate health inequality index, the spatial change over time is rather positive. Out of the total number of LAU1s, 14 (18%) experienced a decrease in the aggregate index over time, while 63 (82%) showed improvement. However, the changes over time are relatively weak, with a difference of 0.023 for the mean and 0.022 for the median between 2016–2019 and 2001–2003. Most LAU1s are situated in the positive portion of the "box" section of the diagram (Fig. 4; Change aggregate index A.1–A.7).

These observations indicate that while the health condition index remained relatively stable, the aggregate index of health inequalities improved, albeit with wider differences between the values. The majority of LAU1s exhibited positive trends in the aggregate index, reflecting some improvements in health inequalities, although these changes were relatively weak.

In this study, we also aimed to assess the influence of our categories of determinants of health inequalities on health indicators, specifically the health condition. We attempted to assess the relationship between health determinants and health indicators (health condition) based on the results of Pearson's correlation coefficient. The results of the correlation analysis using the Pearson correlation coefficient between categories A.1 to A.7 and category B.1 Health condition are as follows (Table 3).

**Table 3:** Pearson correlation coefficient between categories A.1 to A.7 and category B.1

Pearson correlation coefficient	**2001–2003**	**2016–2019**
A.1 Economic status and social protection	0.55	0.70
A.2 Education	0.55	0.62
A.3 Demographic situation	-0.13	-0.34
A.4 Environmental status	-0.10	-0.11
A.5 Individual living status	-0.18	-0.13
A.6 Road safety and crime	0.22	0.43
A.7 Sources of health and social care	0.04	-0.11

**Fig. 5:**
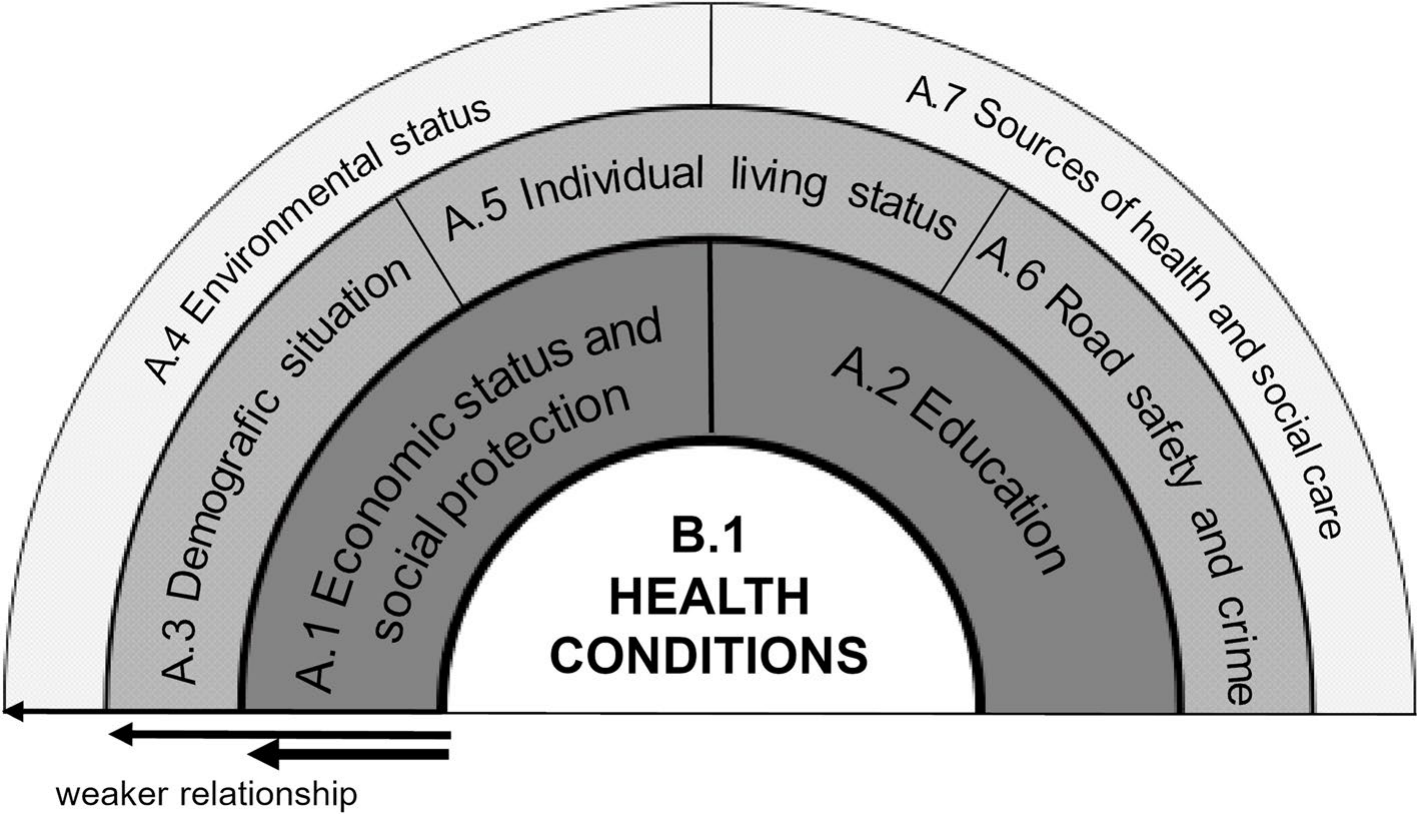
A model of the holistic concept of inequalities of determinants in health (inspired and adapted from [19])

These results are in line with previously published classifications of health determinants and their impact on population health [1, 19]. Building upon the original concept of a classification that focuses on health determinants [10, 13, 19], we used our findings to develop a schematic model illustrating the holistic concept of inequalities in health determinants (see Fig. 5).

**Fig. 6:**
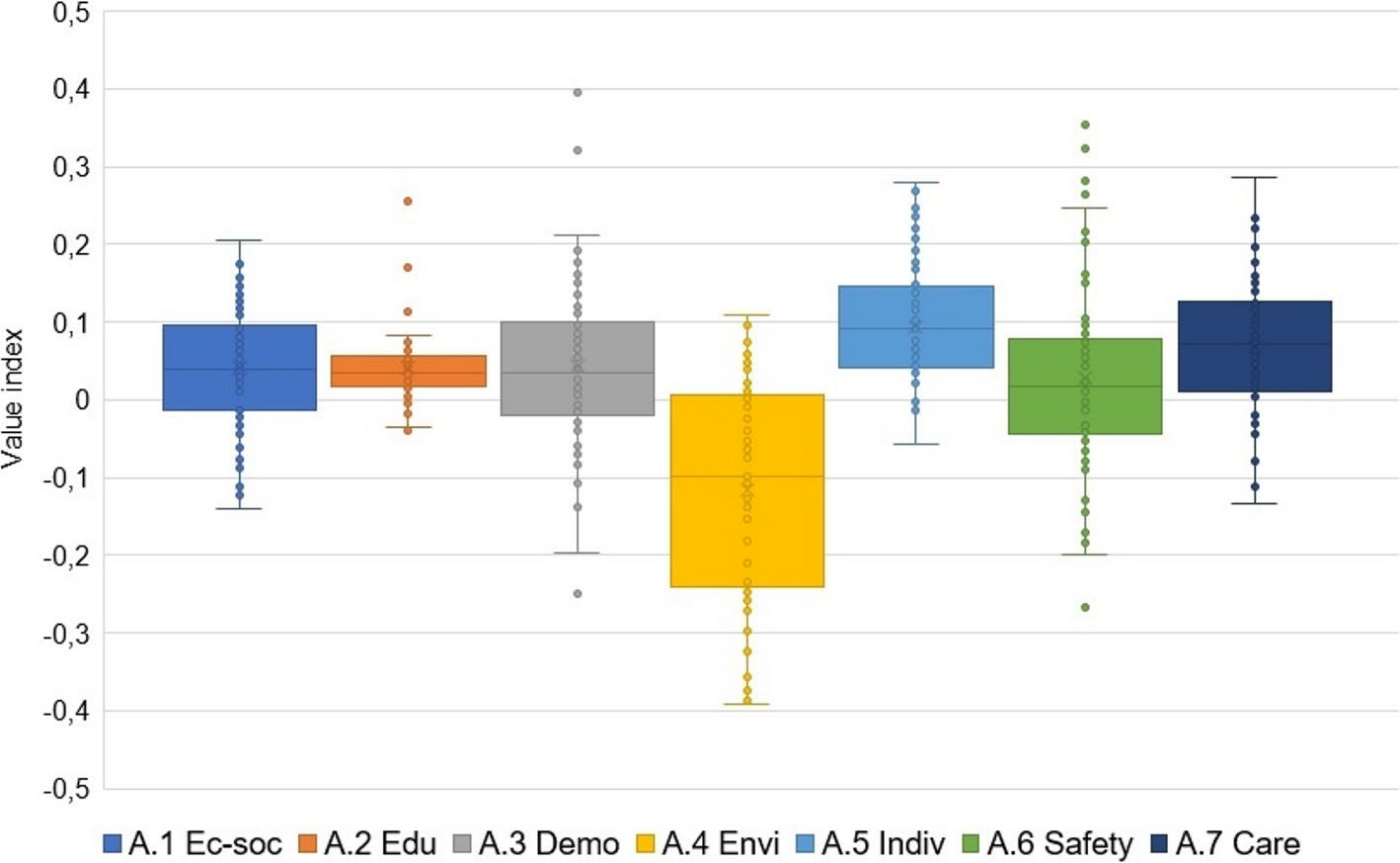
Change in indices of categories of determinants of health inequalities in comparison 2001–2003 and 2016–2019

When comparing the values of individual indices over time in aggregates of all LAU1 between 2001–2003 and 2016–2019, different trends emerge. The majority of categories have shown improvement over time, as indicated by positive values in the middle "box" section of the chart. However, there are noteworthy outliers in the case of A.3 Demographic situation and A.6 Road safety and crime (Fig. 6).

**Fig. 7:**
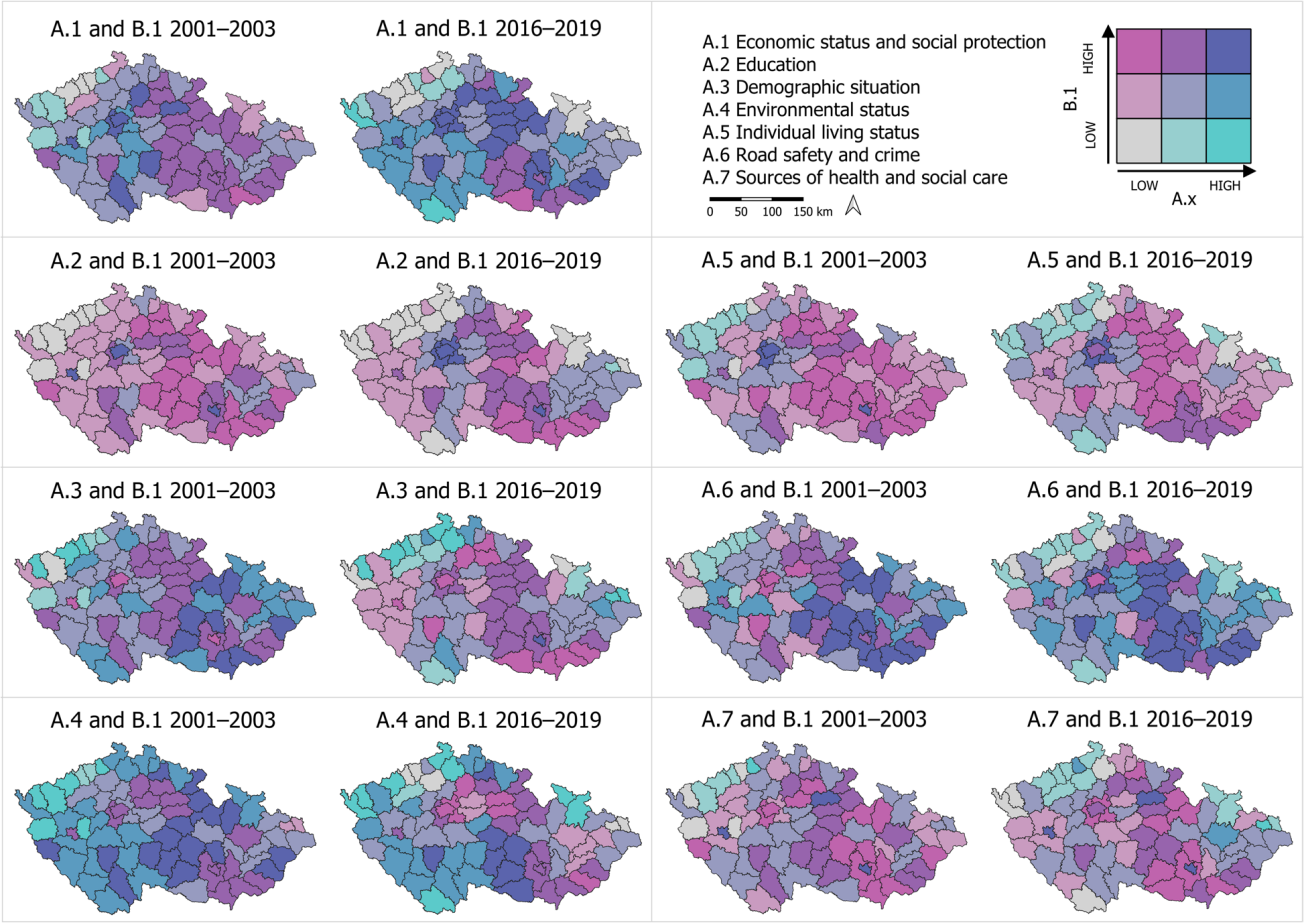
Combination of health inequality category with health condition

We once again utilize visualizations to assess spatiotemporal changes. Our assessment focuses on two aspects: i) Examining how the values of the indices for individual categories of health inequalities (A.1 to A.7) in combination with Health condition (B.1) change over time, comparing the two periods (Fig. 7). ii) Analyzing how the proportion (%) of LAU1 regions out of the total of 77 changes over time in relation to the values of the indices for categories of health inequalities (A.1 to A.7) with Health condition (B.1) (Table 4). It was confirmed that A.1 Economic status and social protection and A.2 Education have the most significant effect on the change of spatial differentiations in the B.1 Health condition index over time. In the group of high values for the category A.1 Economic status and social protection, along with Health condition, the proportion of LAU1 regions increases from 9.1% (of all regions, *n* = 77) in the period 2001–2003 to 22.1% in 2016–2019. In the low-value group for the category A.1 Economic status and social protection, along with Health condition, there is a negative increase from 3.9% to 10.4% of all LAU1 regions. Similarly, for the category A.2 Education, in the high-value group, the proportion increases from 3.9% to 5.2% of LAU1 regions, while in the low-value group, the negative change is more significant, increasing from 10.4% to 16.9%. Regarding the categories A.3 Demographic situation and A.4 Environmental status, the main observation is the decrease in the proportion of positively assessed regions (from 10.4% to 1.3%; and from 22.1% to 9.1%, respectively). Additionally, for category A.4, the proportion of regions with both low value in this category and low Health condition increases (from 0.0% to 6.5%). In the other categories, there was only one positive change, for A.6 Road safety and crime (from 16.9% to 20.1%). The results show that the values of the categories of determinants of health inequalities improve over time (see Fig. 7, Table 4).

**Table 4:** Shares in the combined indices of the categories of determinants of health inequalities and health condition

	Share of LAU1 in 2001–2003 and 2016–2019; %, n = 77							
	High value category and health condition		High category value and low health condition		Low category value and health condition		Low category value and high health condition	
	2001–2003	2016–2019	2001–2003	2016–2019	2001–2003	2016–2019	2001–2003	2016–2019
A.1 Economic status and social protection	9.1	22.1	0.0	2.6	3.9	10.4	1.3	1.3
A.2 Education	3.9	5.2	0.0	0.0	10.4	16.9	24.7	10.4
A.3 Demographic situation	10.4	1.3	3.9	9.1	1.3	2.6	3.9	13. 0
A.4 Environmental status	22.1	9.1	7. 8	10.4	0.0	6.5	0.0	9.1
A.5 Individual living status	3.9	2.6	0.0	0.0	2.6	5.2	7.8	22.1
A.6 Road safety and crime	16.9	20.1	0.0	1.3	2.6	3.9	5.2	2.6
A.7 Sources of health and social care	3.9	3.9	1.3	1.3	5.2	5.2	16.9	14.3

For each of the individual categories A.1 to A.7, we selected the percentages (%) of LAU1 regions with high and low values of the indices for both periods. The same procedure was applied for the Health condition index B.1. The selection of "high" and "low" values was based on data visualization, where these values were determined using natural interval calculation (using QGIS). Specifically, the selected regions always correspond to the LAU1 regions in Fig. 7, represented in the legend by gray (low) or dark blue (high) color. Among the defined categories, A.2 Education, where a low value indicates a low health condition, and A.1 Economic status and social protection, where both high and low values are associated with high and low health condition, play crucial roles. However, the impact of A.4 Environmental status on health condition has diminished over time, particularly for high category values. In the case of A.3 Demographic situation and A.5 Individual living status, the effect on health condition is not significant, as some regions may experience an improvement in health condition despite a decrease in their category values. The influence of A.6 Road safety and crime and A.7 Sources of health and social care on health condition remains relatively unchanged in the area (Table 4).

An intriguing finding arises when comparing changes in the "opposite" relationships between determinant categories and health condition. While the proportion of LAU1 regions with a high category value and low health condition is minimal to zero, and the change over time is insignificant, there are more significant changes observed in the combination of a low category value with a high health condition. In these relationships, the proportion of such LAU1s increased for categories A.3, A.4, and A.5, and decreased notably for category A.2, with a lesser extent for A.6 and A.7 (in Table 4, columns on the right).

**Fig. 8:**
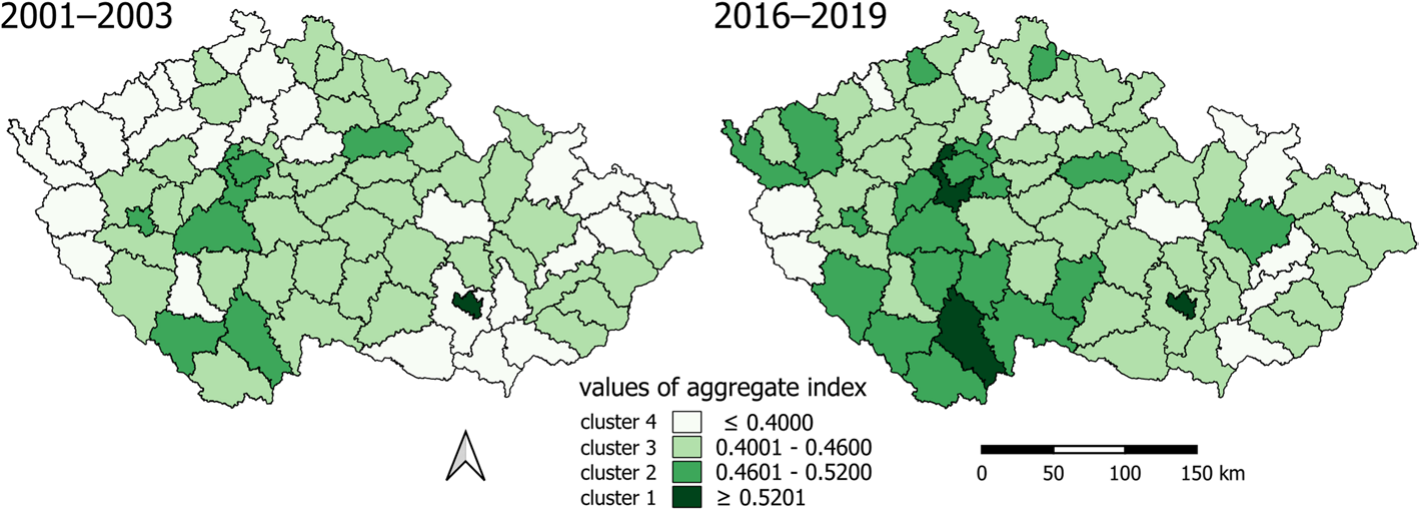
Clusters by aggregate index of determinants of health inequalities

The clusters formed based on the aggregate index of health inequality determinants (from categories A.1 to A.7) delineate geographical regions (Fig. 8).

The comparison of spatiotemporal distribution of clusters reveals a favorable change: the proportion of areas with a lower, rather negative value of the aggregate index (light color, clusters 3 and 4) has decreased, while the proportion with a higher, rather positive value (darker color, clusters 1 and 2) has increased:

- Cluster 4 exhibits the most pronounced health inequalities. In the period 2001–2003, LAU1 consisted of an urbanized outer periphery and rural inner and outer peripheries. The outer urbanized periphery, particularly in the northwest and northeast, experienced economic development during the industrialization era but now suffers from structural unemployment. The border periphery, settled after World War II, faces below-average social capital, economic challenges, and selective migration, which contribute to social exclusion. A positive finding is a significant decrease in urbanized LAU1s in the outer periphery in 2016–2019.
- In contrast, causes of inequality in the rural periphery, whether external or internal, primarily stem from demographic and institutional factors and an inadequate labor market [69]. The number of these LAU1s also declined in 2016–2019.
- Cluster 3 is characterized by significant health inequalities. In the period 2001–2003, it mainly comprises the rural inner periphery, primarily in the central part of the country, and the outer periphery. In 2016–2019, LAU1s from the urbanized outer periphery were added, transitioning from Cluster 1 with the highest health inequalities.
- Cluster 2 exhibits more moderate health inequalities. In the period 2001–2003, it consists of a relatively small number of LAU1s, representing developing regions with various-sized cores (cities as NUTS3 and LAU1 centers) and their surrounding areas. Regions in the hinterland of cities are characterized by the diversification of the Czech countryside, experiencing intensive suburbanization, often serving as migratory-income regions with natural population growth, low unemployment rates, and above-average educational attainment. A positive spatiotemporal change is the increase of these regions in Cluster 3 in 2016–2019.
- Cluster 1 is characterized by the smallest health inequalities but also includes the fewest LAU1s. It comprises highly urbanized LAU1s, with one additional LAU1 in 2016–2019 as a suburban rural area.

**Fig. 9:**
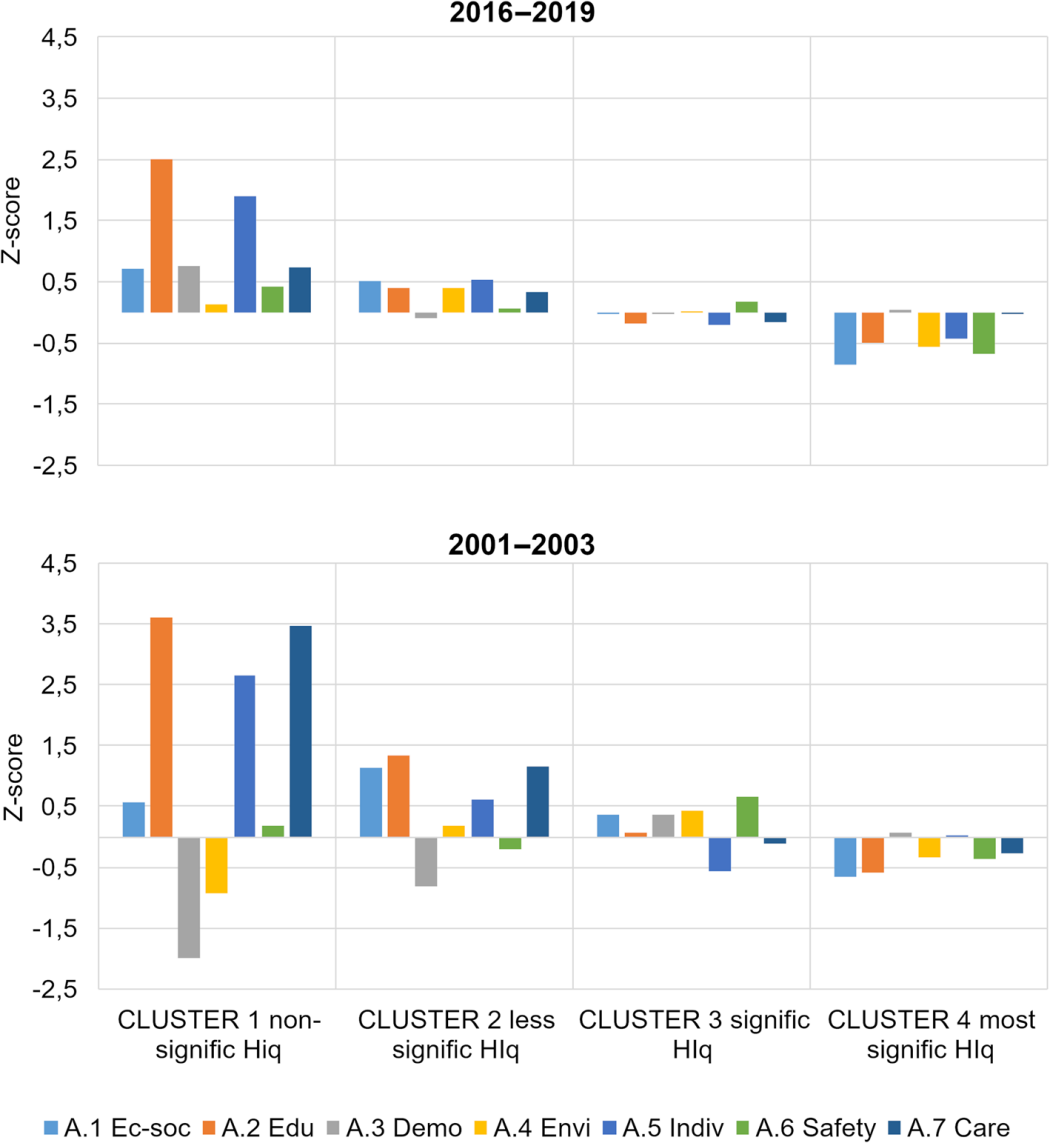
Mean z-score for each of the variables within the clusters

The clusters formed reveal various types of health inequalities and degrees of disadvantage that evolve over time (Fig. 9). A negative value in the z-score of a category signifies a disadvantage or inequality that is challenging to mitigate through other means, given the multidimensional nature of health inequalities and the interplay of determinants [70].

Across all clusters, there has been a moderation of differences in the values of categories related to determinants of health inequalities over time. In clusters 1 and 2, which represent areas with milder inequalities, almost all categories have values in the positive range, indicating an above-average condition. The main improvement in these clusters has occurred in category A.3 Demographic situation. In cluster 3, characterized by more pronounced inequalities, all categories fall around the average values in both periods. Cluster 4, which exhibits the largest health inequalities, shows a stable condition over time, with negative or below-average values.

## Discussion

Leaving aside the genetic basis, environmental, and healthcare factors, socioeconomic factors have a significantly greater influence on health [21, 22]. Our results are consistent with previously published classifications of health determinants and their impact on population health [1, 19]. The strongest relationships between the categories of determinants of health inequalities and Health condition were observed in A.1 Economic status and social protection [27, 37, 68, 70, 71], and A.2 Education [32, 38, 39, 66]. Based on our findings, we developed a schematic model illustrating the holistic concept of health inequalities determinants. This concept builds upon the original classification that focuses on health determinants [10, 13, 19].

In comparison to epidemiological studies, quantifying the influence of the geographic environment on health inequalities proves challenging due to the identification of numerous determinants with spatial characteristics [13]. The clusters in cluster analysis align with the concept of peripheries and cores in the Czech Republic. However, characterizing the typical geographic features of clusters in the Czech environment presents difficulties, given the country's specificities in periphery classification. The Czech periphery encompasses both urban and rural regions, requiring a distinction between inner and outer peripheries. Nevertheless, we can assert that the geographic features correspond to fundamental theories of periphery delineation, albeit with consideration of Czech peculiarities. Economic, social, and societal inequalities observed between peripheries and core areas exhibiting high economic performance in the Czech Republic align with the theory of geographical polarization. In the periphery, additional causes of inequality arise alongside economic challenges, as postulated by the theory of cumulative causes [72]. The rural periphery's current state is shaped by societal and local processes, as well as the transition to post-industrialization, which resonates with the theory of rural restructuring [73].

The spatial inequalities in health and their consistent trends over time indicate that these inequalities do not occur randomly [3]. They reflect the uneven distribution of health risks within the context of geographical characteristics. The combined influence of economic, social [26, 27, 29], and environmental factors [44, 50, 51], along with the availability of local health [60, 67] and social care [7, 61], contribute to regional health disparities. At the spatial scale, both the inner and outer peripheries of LAU1 in the Czech Republic exhibit poorer performance, although it is necessary to differentiate between urban and rural peripheries. The assumption that urban areas perform better than rural areas does not hold true here [13], although improvements have been observed over time in the urbanized periphery. It is positive that when comparing data from the periods 2001–2003 and 2019–2019, regional health inequalities are decreasing. The share of LAU1 areas with a lower (negative) value of the overall health inequality index is declining, while the share with a higher (positive) value is increasing. The largest health inequalities are observed in LAU1 areas located in urbanized outer peripheries and in rural inner and outer peripheries. Urbanized peripheries suffer primarily from structural unemployment and all its consequences. In rural peripheries, the main issues are related to demographic and institutional factors, as well as insufficient labor market opportunities [74]. Additionally, unsatisfactory transportation accessibility, civic amenities, and infrastructure [75], along with selective loss migration, which may exacerbate population aging and unfavorable educational structures, and weak social and cultural capital, are contributing factors [76, 77]. The economic, social, and demographic situation in "peripheral rural areas" is based on processes related to labor market development, land use, construction intensity, and property prices [78].

The combination of poverty and other vulnerability indicators such as age (children, elderly), health disabilities, or minority backgrounds can further amplify these inequalities [71]. Despite the Czech Republic's relative demographic, social, economic, and ethnic homogeneity, and its low proportion of socially excluded individuals or those living below the poverty line compared to other EU countries, it appears that (micro)regional health inequalities persist in the long term [2]. Our results, however, demonstrate that there has been an improvement in the status of most determinants of health inequalities. Despite this improvement, the Czech Republic is not as successful in reducing mortality rates and lowering the intensity of mortality, which are factors influencing the resulting Health Condition Index [68, 70].

Inequalities in health encompass multiple dimensions, including the number of determinants (categories) and their spatiotemporal aspects [13, 25, 79]. In our comprehensive study, we highlight the necessity of interdisciplinary collaboration across various fields such as medicine, sociology, economics, environmental science, and more to address health inequalities effectively. Only through multi-sectoral collaboration can we devise optimal measures that lead to improvements and strengthen policies based on objective and relevant evidence [1]. This collaborative approach is arguably a perspective capable of comprehensively analyzing and tackling 21st-century health challenges [80]. Eliminating or at least mitigating the consequences of health inequalities is not solely an individual concern but, more importantly, a policy issue [3] hat extends beyond the realm of public health [81].

### Limitations of the study

Geographical contexts can be measured and spatial indices constructed differently across various studies, leading to variations in methodological approaches and geographical frameworks. As a result, the comparability of results becomes limited [82]. To address this, we deliberately utilized reliable and publicly available data that present fewer methodological challenges and offer detailed geographical and demographic information. The selection of determinants was guided by the need for applicability and adaptability of methods at international, national, and local levels. However, we acknowledge certain data limitations, particularly in terms of comparability over longer time series. For instance, air quality monitoring in the Czech Republic has undergone significant improvements and refinements since 2001. The original nine monitoring stations that measured benzo[a]pyrene have now been expanded to 46 stationary stations, complemented by mobile stations. Consequently, there has been a perceived deterioration in the benzo[a]pyrene indicator over time. This change can be attributed to the enhanced monitoring coverage of local heating sites and long-range transmission, resulting in more accurate data for interpolation purposes.

## Conclusion

The outcomes of our study can serve as valuable tools for health policy-making and government decision-making. They support targeted actions to eliminate health inequalities and enhance the health of all population groups, aligning with the adopted Strategic Framework for Healthcare Development in the Czech Republic until 2030. The enhancement of the health of the Czech population should be particularly achieved by providing strategic and conceptual support for lifelong prevention.

The regional dimension of the study also holds significant advantages. Within this context, the results are beneficial as a basis for developing regional health policy concepts or formulating documents at the level of the Czech Republic's regions (NUTS3). By acknowledging regional disparities and the multifaceted causes of health inequalities, the translation of results into tools for precise, specialized prevention in public health becomes possible. Additionally, these results bolster and motivate individual prevention efforts.

The implemented measures should aim to nurture a social and physical environment that fosters health, improves the quality of life, and promotes health-promoting behaviors throughout all stages of life. The prioritized measures include supporting the network of regional health centers and exerting a positive influence on key socio-economic determinants. These determinants encompass reducing poverty, especially among seniors, decreasing unemployment in structurally disadvantaged regions and peripheral areas, fostering regional social cohesion, and enhancing healthcare accessibility. Our results support measures defined by the Strategic Framework that focus not only on supporting education in disease prevention but on introducing health education in both primary and secondary schools, and enhancing health literacy across the Czech population.

The implementation of these objectives and measures necessitates optimizing and integrating the core functions of the healthcare system while fostering collaboration with other non-medical disciplines. By doing so, we can aspire to longer lives free from health limitations and major preventable illnesses.

### Supplementary Information

**Additional file 1: Appendix 1. **List of determinants of health.

## Data Availability

All data presented in this study are available from official data sources, namely the Czech Statistical Office, the Institute of Health Information and Statistics of the Czech Republic, the Ministry of Labour and Social Affairs, and the Czech Hydrometeorological Institute. The descriptions of all determinants within each category are included in the Appendix 1. The dataset supporting the conclusions of this article is available in the Zenodo repository, https://doi.org/10.5281/zenodo.8033298.

## Abbreviations

LAU: Local Administrative level
NUTS: Nomenclature of territorial units for statistics
RDS CZ 2021 +: Regional Development Strategy of the Czech Republic 2021 +
MoLSA: Ministry of Labour and Social Affairs
CZSO: Czech Statistical Office
CHMI: Czech Hydrometeorological Institute
IHIS: Institute of Health Information and Statistics of the Czech Republic
WSA: Weighted Sum Approach
LISA: Local Indicator of Spatial Association
