# Supplementary material for: A holistic model of health inequalities for health policy and state administration: a case study in the regions of the Czech Republic
Source: Int J Equity Health. 2023 Sep 5;22:183. doi: 10.1186/s12939-023-01996-2 (PMC10481637; doi:10.1186/s12939-023-01996-2)
Supplement: Supplementary file 1 — Additional file 1: Appendix 1. List of determinants of health. [file 12939_2023_1996_MOESM1_ESM.docx]

**Appendix 1. List of determinants of health**

| **Category** | **Theme** | **Description** | **Year** | **Source** |
| --- | --- | --- | --- | --- |
| **A.1  Economic conditions and social protection** | Employment rate | **Unemployed persons (total):** number of available jobseekers aged 15-64 compared to persons of the same age; % | 2003  2019 | [MoLSA](https://www.mpsv.cz/web/en) |
|  |  | **Unemployed persons (men):** number of available jobseekers / men aged 15-64 compared to persons of the same age; % | 2003  2019 | MoLSA |
|  |  | **Unemployed persons (women):** number of available jobseekers / women aged 15-64 compared to persons of the same age; % | 2003  2019 | MoLSA |
|  |  | **Jobseekers aged 50-64:** number of jobseekers aged 50-64 compared to people of the same age; % | 2003  2018 | [CZSO](https://www.czso.cz/csu/czso/home) |
|  |  | **Jobseekers aged 15-24:** number of jobseekers aged 15-24 compared to people of the same age; % | 2003  2018 | CZSO |
|  |  | **Jobseekers with an unemployment duration of 12 months or more:** number of jobseekers with an unemployment duration of 12 months or more compared to the total number of jobseekers; % | 2003  2018 | CZSO |
|  |  | **Jobseekers with basic education:** number of jobseekers with basic education compared to the total number of jobseekers; % | 2003  2018 | CZSO |
|  |  | **Jobseekers with secondary vocational education without completing a leaving exam (incl. apprentices):** number of jobseekers with secondary vocational education without completing a leaving exam (incl. apprentices) compared to the total number of jobseekers; % | 2003 2018 | CZSO |
|  | Living conditions and social benefits | **GDP/capita:** share of GDP per capita; and 100 thou. CZK; REGIONS (identical value of the region level assigned to the districts of the respective region) | 2001 2018 | CZSO |
|  |  | **Housing allowances:** total amount of housing allowances for persons aged 20 and over; in CZK/person | 2003 2018 | CZSO |
|  |  | **Child allowances:** total amount of child allowances for persons aged 20-59; in CZK/person | 2003 2018 | CZSO |
| **A.2  Education** | Educational structure | **Population with basic incl. incomplete education:** share of persons with basic and incomplete education aged 15 and over in relation to persons of the same age; % | 2001  2011 | CZSO |
|  |  | **Population with university education:** share of persons with university education aged 15 and over in relation to persons of the same age; % | 2001  2011 | CZSO |
| **A.3 Demographic situation** | Migration | **Foreigners by most frequent citizenships:** sum of the number of most frequent citizenships of foreigners in relation to the whole population; % | 2001 2019 | CZSO |
|  | Aging | **Age index:** number of persons aged 65 and over compared to the number of persons aged 0-14; % | 2001 2019 | CZSO |
|  | Urbanisation | **Level of urbanisation:** share of population living in cities; % | 2003 2019 | CZSO |
| **A.4  Environmental status** | Air quality | **Five-year average of annual average concentrations of suspended particulate matter PM_2.5_;** µg.m^-3^ | 2007 2018 | [CHMI](https://www.chmi.cz/?l=en) |
|  |  | **Five-year average of annual average concentration of suspended particulate matter PM_10_;** µg.m^-3^ | 2007 2018 | CHMI |
|  |  | **Five-year average of annual average concentration of benzo[a]pyrene;** ng.m^-3^ | 2007 2018 | CHMI |
|  |  | **Five-year average of annual average NO_2_ concentration;** µg.m^-3^ | 2007 2018 | CHMI |
|  |  | **Five-year average of annual average benzene concentration;** µg.m^-3^ | 2007 2018 | CHMI |
|  | Countryside | **Coefficient of ecological stability KES:** share of ecologically stable areas to unstable areas; coefficient | 2003 2019 | CZSO |
| **A.5 Individual living status** | Living conditions | **Average living space per person;** m^2^ | 2003 2011 | CZSO |
|  |  | **Heating method:** share of dwellings heated by electricity or gas to dwellings heated by solid fuels; coefficient | 2001 2011 | CZSO |
|  | Technical infrastructure | **Share of municipalities in the district with connection to the sewerage system terminated by a WWTP;** % | 2000 2016 | CZSO |
| **A.6  Road safety and crime** | Traffic accidents | **Total traffic accidents:** total number of traffic accidents in relation to the total population; per 1 thousand inhabitants | 2003 2018 | CZSO |
|  |  | **Traffic accidents under the influence of alcohol:** number of traffic accidents under the influence of alcohol relative to the total population; per 1 thousand inhabitants | 2003 2018 | CZSO |
|  |  | **Deaths due to road accidents:** number of deaths due to road accidents relative to the number of inhabitants (ESP2013); per 100 thousand inhabitants | 2001  2017 | [IHIS](https://www.uzis.cz/index-en.php) |
|  | Crime | **Deaths due to assault (attack):** number of deaths due to assault (attack) relative to number of inhabitants (ESP2013); per 100 thousand inhabitants | 2001  2017 | IHIS |
|  |  | **Registered offenses:** number of registered offenses in relation to the total population; per 1 thousand inhabitants | 2003  2018 | CZSO |
| **A.7 Sources of health and social care** | Health and social care capacities | **Physicians in healthcare facilities:** number of physicians relative to the total population; per 1 thousand inhabitants | 2003  2018 | CZSO |
|  |  | **Hospital beds:** number of hospital beds in relation to the total population; per 1 thousand inhabitants; REGIONS (identical value of the region level assigned to the districts of the respective region) | 2003  2018 | CZSO |
|  |  | **Places in social services facilities:** number of places in social services facilities relative to the total population; per 1 thousand inhabitants; REGIONS (identical value of the region level assigned to the districts of the respective region) | 2003  2018 | CZSO |
| **B.1  Health condition** | Life expectancy and mortality structure | **Life expectancy at birth men** | 2001  2019 | CZSO |
|  |  | **Life expectancy at birth women** | 2001  2019 | CZSO |
|  |  | **Life expectancy at age 65 men** | 2001  2019 | CZSO |
|  |  | **Life expectancy at age 65 women** | 2001  2019 | CZSO |
|  |  | **Total mortality:** total number of deaths relative to total population (ESP2013); per 100 thousand inhabitants | 2001  2017 | IHIS |
|  |  | **Male mortality:** number of male deaths relative to total male number (ESP2013); per 100 thousand inhabitants | 2001  2017 | IHIS |
|  |  | **Female mortality:** number of female deaths relative to total women (ESP2013); per 100 thousand inhabitants | 2001  2017 | IHIS |
|  |  | **Infant mortality:** number of deaths within 1 year relative to the total number of live births; per 1 thousand live births | 2001  2018 | CZSO |
|  |  | **Neonatal mortality:** number of deaths within 28 days of birth versus number of live births; per 1 thousand live births | 2003  2018 | CZSO |
|  |  | **Deaths from infectious and parasitic disease :** number of deaths from infectious and parasitic diseases relative to total population (ESP2013); per 100 thousand inhabitants | 2001  2017 | IHIS |
|  |  | **Deaths from circulatory system diseases:** number of deaths from circulatory system diseases relative to the total population (ESP2013); per 100 thousand inhabitants | 2001  2017 | IHIS |
|  |  | **Deaths from respiratory diseases:** number of deaths from respiratory diseases relative to the total population (ESP2013); per 100 thousand inhabitants | 2001  2017 | IHIS |
|  |  | **Deaths from malignant neoplasms:** number of deaths from malignant neoplasms relative to the total population (ESP2013); per 100 thousand inhabitants | 2001  2017 | IHIS |
|  |  | **Deaths from gastrointestinal diseases:** number of deaths from gastrointestinal diseases relative to the total population (ESP2013); per 100 thousand inhabitants | 2001  2017 | IHIS |
|  |  | **Deaths from other causes:** number of deaths from other causes relative to the total population (ESP2013); per 100 thousand inhabitants | 2001  2017 | IHIS |
|  |  | **Intentional self-harm men:** number of men who died as a result of self-harm relative to the total number of men (ESP2013); per 100 thousand inhabitants | 2001  2017 | IHIS |
|  |  | **Intentional self-harm woman:** number of women who died as a result of self-harm relative to the total number of women (ESP2013); per 100 thousand inhabitants | 2001  2017 | IHIS |
|  |  | **Deaths due to liver disease:** number of deaths from liver disease (alcoholic, toxic, cirrhosis, chronic and other inflammations and diseases) relative to the total population (ESP2013); per 100 thousand inhabitants | 2001  2017 | IHIS |
|  |  | **Deaths due to smoking tobacco:** number of deaths due to smoking tobacco (malignant neoplasm of larynx, trachea, bronchi and lungs) relative to the total population (ESP2013); per 100 thousand inhabitants | 2001  2017 | IHIS |
|  | Abortion rate | **Spontaneous abortion index:** number of spontaneous abortions versus number of live births; per 100 live births; % | 2001  2017 | CZSO |
|  |  | **Induced abortion index:** number of abortions versus number of live births; per 100 live births; % | 2001  2017 | CZSO |
|  | Diabetes | **Treated diabetics:** number of diabetics treated relative to the total population; per 100 thousand inhabitants | 2003  2016 | CZSO |
|  |  | **Diabetes mellitus deaths:** number of deaths due to diabetes mellitus relative to the total population (ESP2013); per 100 thousand inhabitants | 2001  2017 | IHIS |
|  | Maturity of a child at birth | **Birth weight:** share of births to a birth weight of 2,500 g relative to all births; per 100 live births; % | 2001  2018 | CZSO |
